# Conditional probability modulates visual search efficiency

**DOI:** 10.3389/fnhum.2013.00683

**Published:** 2013-10-17

**Authors:** Bryan Cort, Britt Anderson

**Affiliations:** ^1^Department of Psychology, University of WaterlooWaterloo, ON, Canada; ^2^Centre for Theoretical Neuroscience, University of WaterlooWaterloo, ON, Canada

**Keywords:** attention, visual search, probability, implicit learning, reaction time

## Abstract

We investigated the effects of probability on visual search. Previous work has shown that people can utilize spatial and sequential probability information to improve target detection. We hypothesized that performance improvements from probability information would extend to the efficiency of visual search. Our task was a simple visual search in which the target was always present among a field of distractors, and could take one of two colors. The absolute probability of the target being either color was 0.5; however, the *conditional probability*—the likelihood of a particular color given a particular combination of two cues—varied from 0.1 to 0.9. We found that participants searched more efficiently for high conditional probability targets and less efficiently for low conditional probability targets, but only when they were explicitly informed of the probability relationship between cues and target color.

## Introduction

The effects of cues in attentional tasks are well documented (Posner and Cohen, 1984; Wright and Ward, 2008; Carrasco, 2011). For the most part, cuing and attention are framed in terms of valid/invalid and present/absent, but such binary characterizations may obscure important distinctions (Anderson, 2011), particularly that attentional effects may operate on a continuum. In addition, it may be that there are more direct accounts for the source of attentional effects that also naturally account for its continuous nature. Probability is one such alternative characterization. In the introduction, we briefly highlight some of the earlier work showing cue effects that vary in proportion to probability information about target features. From these data, we hypothesize that the efficiency of search should also improve when cues communicate a target's probable features.

As far back as 1980, it has been recognized that cuing is not an all or none process and that cue effects are graded by validity (Jonides, 1980; Eriksen and Yeh, 1985; Madden, 1992; Riggio and Kirsner, 1997). Jonides (1980) employed a circular 8-item search display in which subjects were shown a neutral, valid, or invalid cue, and the predictive value of the valid cue varied between 30, 50, and 70%. The magnitudes of reaction time (RT) cost (for invalid cues) and benefit (for valid cues) increased in proportion to the validity of the cue. Eriksen and Yeh (1985) presented subjects with an identical display and varied the predictive value of both a primary spatial cue and a secondary spatial cue, and also found that RTs improved in proportion to the predictive value of the cues. Many of these data are well described by a Bayesian characterization of visual search (Vincent et al., 2009; Ma et al., 2011).

The last decade has seen an increase in interest on this topic. Vossel et al. (2006) collected fMRI and RT data in a slight variation of Posner's (Posner et al., 1980) seminal cuing task. They varied cue validity between a 60% condition and a 90% condition, confirming the finding that greater cue validity resulted in faster RTs and showing that cue validity modulates activation in a right-hemispheric fronto-parietal attentional network. In a similar design, Gould et al. (2011) also demonstrate such a relationship between cue validity and RT. Hahn et al. (2006) demonstrated probability spatial cuing effects in a simple search task. Targets occurred at one of four peripheral locations, and a central symbolic cue indicated which quadrants were of greater probability for a particular trial. Any number (up to all four) of the quadrants could be cued on any given trial, and the cue validity was 80%, regardless of the number of quadrants cued. This yielded a graded cue validity ranging from 25% (all quadrants cued) to 80% (one quadrant cued). The primary behavioral result was a monotonic relationship between the number of primed positions and RT, with fewer primed locations (and thus greater cue validity) generating faster RTs. These results both reinforce and affirm probabilistic effects in attentional tasks with more complex methods (serial search) and modern methodologies (fMRI).

The vast majority of work on graded cuing effects has involved spatial cues. To our knowledge, there is only one investigation of graded effects of feature cue validity. Egner et al. (2008) explored spatial and feature cuing when the predictive value of the cues was parametrically varied. They utilized a simple search task in which a fixed grid of four locations contained diamonds that were either red or blue in color and left or right in spatial position. Central cues communicated independent information about location and color of the target. The validity of the spatial and color cues was 50, 70, or 90%, and the probability that any particular diamond was the target was the product of the individual cues. The task required the participants to locate the target diamond, which was distinguished by a missing corner. The principle result was a relationship for cue predictive value and RT. Trials with 90% valid cues were faster than 70% valid cue trials, and 90% invalid cue trials (or alternatively, 10% predictive value) were slower than 70% invalid cue trials (alternatively, 30% predictive value). There was no significant effect of cue dimension, meaning that both spatial and feature cues had equivalent effects. The relationship was non-linear because the magnitude of cuing effects between 70 and 90% was less than that for 50 and 70%. The magnitude of each cue was greatest when the other was non-informative (50% predictive value). While this study demonstrates attentional effects for probability cues (color) it did not vary the number of display elements, thus leaving open the question of whether probability cuing also affects the efficiency of search.

When tested, the relationship between attention and cue predictive value is found to be graded rather than all or none. What defines a cue's predictive value is its probabilistic relation to the target. This asserts an equivalence between cues and prior probability. There is a great deal of evidence that such statistical relationships can be learned implicitly and on line (Saffran et al., 1996, 1997, 1999; Chun and Jiang, 1998, 1999; Chun, 2000; Fiser and Aslin, 2001, 2002; Geng and Behrmann, 2002, 2005; Jiang and Leung, 2005; Ono et al., 2005; Williams et al., 2009; Druker and Anderson, 2010; Jiang et al., 2013).

We are aware of comparatively few studies that have investigated non-spatial aspects of probability learning. Chun and Jiang (1999) implemented a variation on their previous (Chun and Jiang, 1998) search task in which instead of searching for a T among L's, participants searched for a shape with a vertical axis of symmetry among shapes with non-vertical axes of symmetry, and they found that distractor identities were effective cues of unique target identity. However, Endo and Takeda (2004) employed a slightly modified version of the same Chun and Jiang (1998) task, in which participants searched for a closed contour among open contours. They found that although target position could be cued both by distractor position and distractor identity, target identity could not be effectively cued by either distractor position or identity. Although the findings of these two studies on statistical learning in search are contradictory, a growing body of work using non-search paradigms suggests that non-spatial probabilities, including feature probabilities similar to those of interest in the current work, can be learned (Fiser and Aslin, 2001, 2002; Kirkham et al., 2002; Turk-Browne et al., 2005, 2008; Baldwin et al., 2008; Brady and Oliva, 2008; Brady et al., 2009). To take an example from the listed works, Fiser and Aslin (2001, 2002) demonstrated that participants can learn joint and conditional probabilities for sequences; though the authors frame this result as learning “groups” of related stimuli independently of order, it can be viewed as one item in the sequence probabilistically cuing the features of a subsequent item. This suggests that the learning of probability relationships need not be limited to spatial tasks.

To test our hypothesis that probabilistic cuing affects search efficiency, we performed four experiments where participants' performed a challenging visual search task. For all experiments participants searched for the one diamond out of several that had one of its four corners missing. They reported which corner was absent. The target diamond was camouflaged by varying numbers of distractor diamonds. In Experiment 1, a combination of two cues provided statistical information about the likely color of the target diamond. To test that any benefits seen in Experiment 1 were not explained by color priming alone, Experiment 2 utilized the same cues and targets, but without any predictable probability information. To examine if it was necessary for the cues and targets to share the salient feature (color in this case) we performed Experiment 3 where one of the cues was made more abstract (present or absent). Lastly, in Experiment 4 we tested whether or not participants had to be explicitly informed of the cue target probability relationship.

## Methods

The methods were similar for all four experiments and are presented here with the salient difference highlighted when the results are presented.

### Participants

Participants in all experiments were University of Waterloo undergraduate students. Experiment 1 had 10 participants (one male, nine females); Experiment 2 had 10 participants (six males, four females); Experiment 3 had 18 participants (four males, 14 females); Experiment 4 had 60 (20 per between subjects condition) participants (25 males, 34 females, one undeclared) of which one was dropped for low accuracy (over 5 SD below the mean). The University of Waterloo Office of Research Ethics approved the research and informed consent was obtained from all participants.

### Procedures

**Figure 1:**
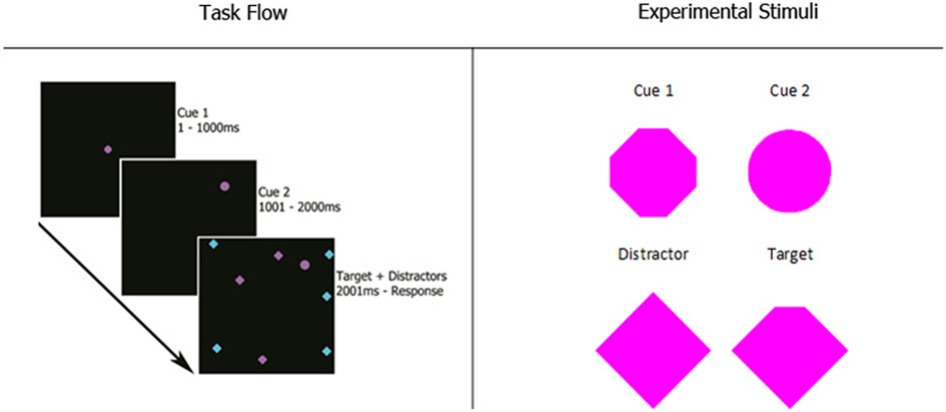
**Experiment 1 task flow and magnified stimuli**. Cue 1 appeared in the center of the screen for 1 s. After it disappeared, cue 2 appeared in a random location. After another 1-s interval, the search array appeared with the target and all distractors in random, non-overlapping locations. Possible stimuli colors were magenta and cyan. The size of every stimulus was 0.8 degrees of visual angle. Participants reported which of the four corners of the target was missing with the corresponding arrow key on the computer keyboard.

Participants completed five blocks of 100 trials of a visual search task. As shown in the first panel of Figure 1, participants viewed two cues in sequence, each for 1000 ms, followed by a search array. Panel 2 gives a detailed view of the experimental stimuli.

The first cue was always in the center of the screen, while the second cue appeared at a random location. During the search task, the second cue remained on the screen and N (8, 12, 16, or 20) items appeared on the screen, all in random locations. The items consisted of a single target, which was a diamond shape with one corner missing, and (*N* − 1) distractors, which were intact diamonds. These items remained until the participant indicated with a button press that they had located the target. After this button press, the search array disappeared and participants were prompted to indicate which corner of the target was missing by pressing the corresponding arrow key on a computer keyboard.

**Figure 2:**
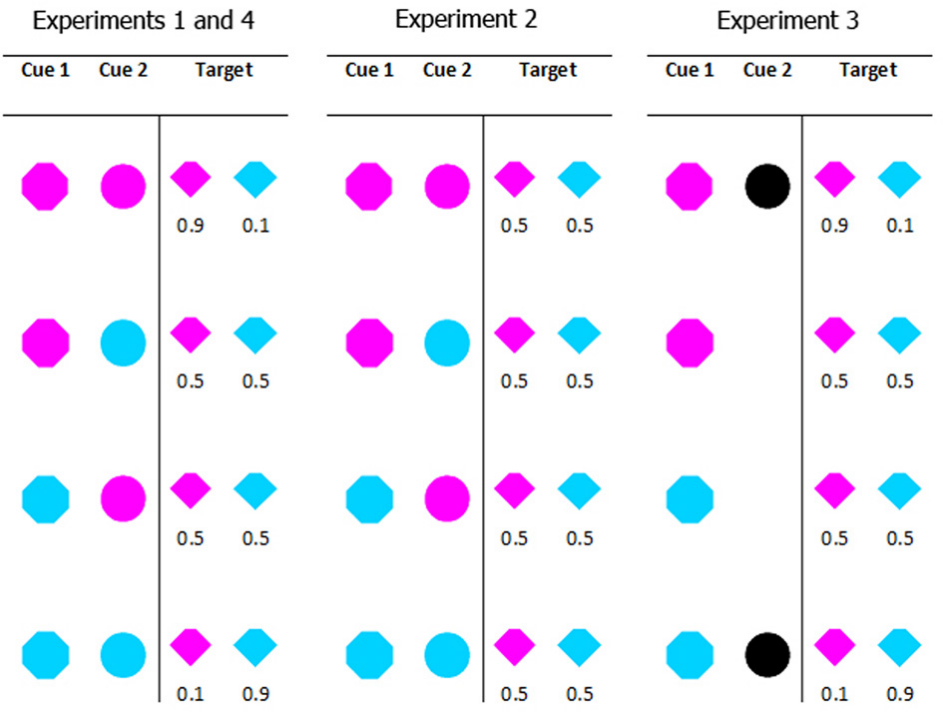
**All possible cue combinations and their associated target color probabilities for each experiment**. Experiments 1 and 4 used the same cue conditions (in Experiment 4 we manipulated the instructions given to participants). In Experiment 1 when both cues were the same color (cyan or magenta) the target was 90% likely to be that color. When the cues were different colors, they were uninformative as to the target color. In Experiment 2, all cue combinations were uninformative. In Experiment 3, the presence or absence (counterbalanced across participants) of cue 2 indicated the validity of cue 1; a valid cue 1 predicted target color with 90% accuracy.

The relation between the cues and target color varied between experiments. In Experiment 1 the absolute probability of a particular color target on any given trial was 0.5; however, the *conditional probability*—the likelihood of a particular target color given a particular cue combination—varied from 0.1 (when both cues predicted the non-target color) to 0.5 (when one cue predicted the target color and the other predicted the non-target color) to 0.9 (when both cues predicted the target color). For example, if both cues were magenta, the target was 90% likely to be magenta and 10% likely to be cyan; if one cue was cyan and the other cue was magenta, the target was 50% likely to be magenta and 50% likely to be cyan. The coloring of the target and distractors was selected on an item by item basis. This means that while the colors cyan and magenta were equally likely overall, on individual trials the number and proportion of each color varied. Panel 1 in Figure 2 shows the full conditional probability distribution for the cues in Experiment 1. In Experiment 2 the same cue color combinations were used, but the conditional probability information was eliminated. In Experiment 3 the probability information was retained, but the second cue was no longer colored. It was either present or absent. The cues and the probability information in Experiment 4 were identical to Experiment 1; only the instructions to participants changed. Figure 2 provides a graphical summary of these details.

For Experiments 1, 2, and 3 participants were explicitly instructed at the beginning of the task about the cue target relationships. As an example, the exact wording used to communicate the relationship between cues and targets in Experiment 1 was, “… if both cues are red, the target is very likely to be red; if both cues are green, the target is very likely to be green; if the cues are each a different color, the target is equally likely to be red or green.” Subjects were told that the second cue would appear in a random position on the screen, and that the location of the second cue was not predictive of any aspect of the search task.

Distractor color ratios were not fixed. Ratios were generated for each trial by assigning each distractor, one by one, a color. This color assignment was weighted by the proportion of colors already assigned to the target and all distractors. The probability of a distractor receiving a certain color was equal to the proportion of stimuli (target + distractors) of the other color. So if there were 3 cyan stimuli and 4 magenta stimuli in an 8-item trial, the last stimulus had a probability of 4/7 to be cyan. This algorithm for generating distractor colors resulted in ratios that converged on 1:1 quite rapidly, and the ratio distribution across all trials was hyper-normal, centered on 1:1.

### Apparatus

Participants sat at a viewing distance of approximately 65 cm from a flat CRT monitor (36.5 × 27.5 cm viewable area, approximately 31° × 24° of visual angle computed at screen center) running at 85 Hz and at 800 × 600 resolution. All stimuli (cues, target, and distractors) subtended 0.8 degrees of visual angle and were presented on a black background. The stimulus presentation program was written in Python and used the Psychopy library (Peirce, 2007).

## Results and discussion

In all experiments, there were no significant effects of target color; magenta or cyan, the RT did not differ. For trials where the two cues were a different color, the order of presentation did not matter. That is, there was no additional benefit or deficit when the second cue color matched the target color compared to when the first cue did. Therefore, all analyses collapse across these factors. This yields a 3 (conditional probability) × 4 (number of stimuli in search display) design. Accuracy in all experiments was near-perfect (98.7% overall; above 98% for each individual experiment) and did not differ between any conditions in any experiments; because of this uniformity, accuracy is not reported below. Analyses used the RTs from correct trials only. As sub-300ms trials had chance levels (33%) of accuracy, we categorized these as accidental button presses and excluded them. Trials over 10 s were more than three standard deviations greater than the mean RT (in all experiments) and we excluded them as reflecting extended periods of off-task behavior. These timing cutoffs resulted in the following proportions of dropped trials: 1.12% in Experiment 1; 1.72% in Experiment 2; 1.14% in Experiment 3; and 2.98% in Experiment 4. At debriefing, no participants reported using any particular search strategy; thus, no participants were dropped from the analysis on this basis for any experiment.

**Figure 3:**
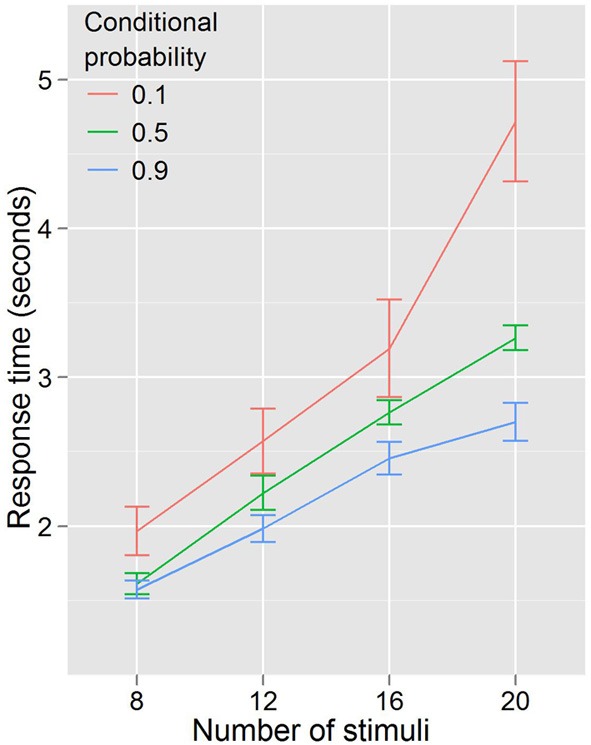
**RTs by distractor number for Experiment 1**. The higher the conditional probability, the faster and more efficient the search.

Experiment 1 showed that RTs were faster for trials where the cues predicted target features and that the RT—stimulus number slope increased different across probability conditions. A repeated measures ANOVA with RT as the dependent variable, and with conditional probability (3 levels: 0.1, 0.5, 0.9) and number of stimuli (4 levels: 8, 12, 16, 20) as factors (Figure 3) found that RT increased with number of stimuli, *F*_(3, 27)_ = 96.29, *p* < 0.001, and decreased with conditional probability, *F*_(2, 18)_ = 18.01, *p* < 0.001. There was also a conditional probability by number of stimuli interaction, *F*_(6, 54)_ = 7.451, *p* < 0.001, indicating that as the number of stimuli increased, the effect of conditional probability became larger. To phrase this result more conventionally, the distractor number × RT slope decreases as conditional probability increases. Distractor number × RT slope is an index of visual search efficiency (Wolfe et al., 1989; Wolfe, 1994, 1998, 2007). Experiment 1 demonstrated that participants are faster to locate and report targets with high conditional probability, and that this change in performance is accompanied by changes in the efficiency of search. One strategy that was suggested as a basis for this result was that participants exhaustively search the cued color first. This is unlikely. First, no participant in this (or any subsequent) experiment reported using such a strategy in the post-questionnaire (in fact, no participants reported using *any* conscious search strategy). Second, RT is better explained by the total number of targets and not just those of the same, high probability color. We formally tested this using linear models (R Development Core Team, 2011). First, we subselected the trials where both cues were of a single color and the target was of the same color. Under the assumption that participants exhaustively search the cued color first, there should be no effect of the number of distractors of the uncued color, because for this subset of trials the participant will never need to search any of them. Two linear models were generated and compared. Model 1 took into account only the number of stimuli on the screen that were of the same color as the target and the cues. Model 2 added as a factor the number of cued-color stimuli and the number of uncued-color stimuli. Table 1 shows the intercepts and coefficients.

**Table 1:** **Intercepts and coefficients for the models generated by simple linear regression on the data from the *0.9 conditional probability* condition in Experiment 1**.

**Model 1: rt = α + β_1_(number cued − color stimuli)**					
	**Value**	***SE***	***df***	***t***	**Significance**
α	1.042	0.086	2199	12.10	*p* < 0.001
β_1_	0.163	0.012	2199	14.11	*p* < 0.001
**Model 2: rt = α + β_1_(number cued − color stimuli) + β_2_(number uncued − color stimuli)**					
α	0.929	0.089	2198	10.448	*p* < 0.001
β_1_	0.113	0.015	2198	7.310	*p* < 0.001
β_2_	0.077	0.016	2198	4.773	*p* < 0.001

Both models demonstrate that as the number of display items of the same color as the target and cues increases so does the RT. Model 2 shows that there is the same relationship for display items of the “wrong” color as well. Since these models are nested we can compare the increase in goodness of fit with a chi-square ratio test (Bentler and Bonett, 1980). This is implemented in R with the ANOVA function (Chambers and Hastie, 1992). Model 1 had an *R*^2^ = 0.083, *F*_(1, 2199)_ = 199.1, *p* < 0.001, and Model 2 has an *R*^2^ = 0.092, *F*_(2, 2198)_ = 111.9, *p* < 0.001. Comparing the models directly verifies the improvement in fit from model 1 to model 2, *F*_(1, 2198)_ = 22.781, *p* < 0.001. Although the improvement in fit is objectively small, the strong correlation between the number of cued and uncued stimuli (*r* = 0.94) leaves very little variance for the number of uncued-color distractors term to explain. That the addition of such a term, with such small explanatory potential, nonetheless results in an improvement in fit provides strong evidence that participants are not simply engaging in an exclusive, exhaustive search of cued-color items.

Although the second cue remains on screen during the search, the models described above do not consider it as a stimulus for two reasons. First, it is different in form from the true distractors; second, it is present on the screen for a full second before the search array is displayed, giving the participant time to recognize and categorize it as not part of the search array. Additionally, in the high conditional probability data, the second cue color always matches the target: so even if participants were responding to the second cue as a distractor during search, it would have no impact on the structure or fit of either model.

**Figure 4:**
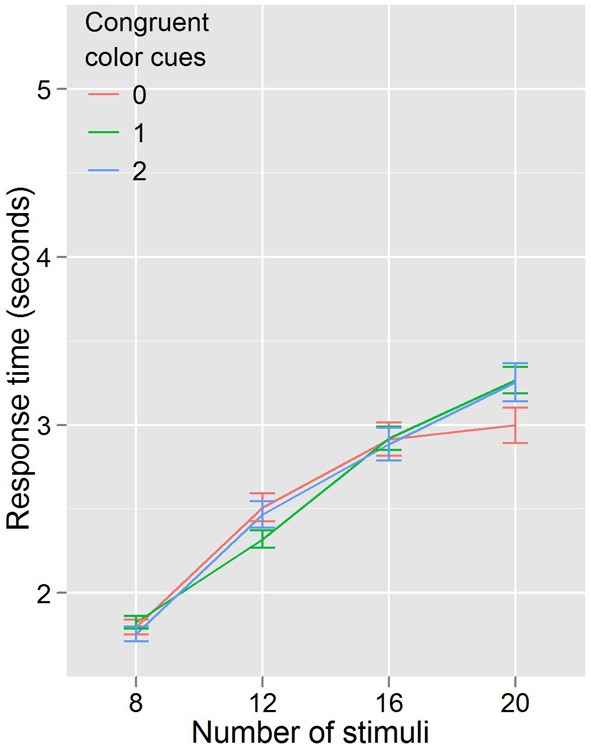
**Response times by distractor number for Experiment 2**. When probability cuing is removed, search efficiency is equal regardless of color priming.

In Experiment 2 we kept the cue and target colors the same, but eliminated the conditional probability information communicated by cue congruence. Thus, if the reduction in RT were completely explained by color priming, that is that two magenta (cyan) targets sped the detection of a subsequent magenta (cyan) target, regardless of probability information, then Experiment 2's results (Figure 4) should look identical with Experiment 1's (Figure 3). As shown in Figure 4 this is not the case, and the lack of an effect of color congruency on RT was confirmed statistically. A repeated measures ANOVA with RT as the dependent variable, and with congruent color cues (3 levels: 0, 1, 2) and number of stimuli (4 levels: 8, 12, 16, 20) as factors revealed a significant effect of the number of stimuli, *F*_(3, 27)_ = 115.587, *p* < 0.001. However, there was no effect of the number of congruent color cues *F*_(2, 18)_ = 0.451, *p* = 0.644, and no number of stimuli by congruent color cues interaction *F*_(6, 54)_ = 1.433, *p* = 0.219. This supports our hypothesis that it is conditional probability, not color priming, driving the effect on search efficiency. However, from the data in Experiments 1 and 2, we still cannot discount the possibility that the conditional probability information is necessary but not sufficient to drive the observed effects on search efficiency; it could be that the combination of color priming and conditional probability is modulating search efficiency. Although this objection seems somewhat more unlikely than that raised with regard to color priming alone, we nonetheless sought to address it in Experiment 3.

Experiment 3 reinstated the manipulation of conditional probability, but the appearance of the cues that delivered the conditional probability information to the participant was changed. In Experiments 1 and 2, the second cue was a circle with two possible states. State 1 (magenta) indicated an increased conditional probability of magenta, and state 2 (cyan) indicated an increased conditional probability of cyan. In Experiment 3, the second cue changed visually to a white circle that was either present (state 1) or absent (state 2). Panel 3 in Figure 2 shows the cues and conditional probability distribution for Experiment 3. In this Experiment 3, cue 2 was indirect and indicated whether the first cue was predictive or non-predictive. This change had two effects: it eliminated half of the color priming from the task, and it made the relationship between cues and target more complicated.

**Figure 5:**
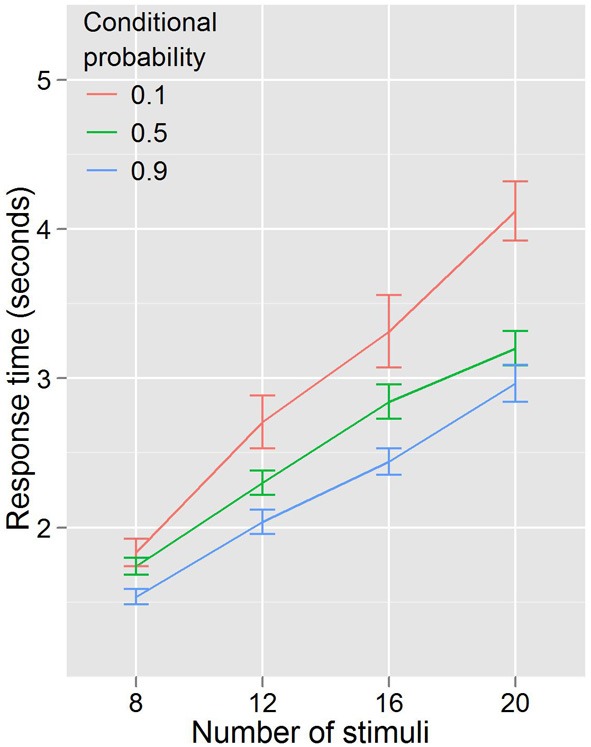
**Response times by distractor number for Experiment 3**. Search efficiency is modulated by conditional probability even when color priming is reduced.

Figure 5 shows that with return of the cue-target probability relationship there was a return to the difference in search slopes. A repeated measures ANOVA with RT as the dependent variable, and with conditional probability (3 levels: 0.1, 0.5, 0.9) and number of stimuli (4 levels: 8, 12, 16, 20) as factors demonstrated a pattern of effects for Experiment 3 identical to Experiment 1; RT increased with number of stimuli, *F*_(3, 51)_ = 158.34, *p* < 0.001, and decreased with conditional probability, *F*_(2, 34)_ = 18.239, *p* < 0.001, and there was a conditional probability by number of stimuli interaction, *F*_(6, 102)_ = 5.458, *p* < 0.001, again showing that the effect of conditional probability was accompanied by changes in efficiency; however, unlike Experiment 1, changes in search efficiency were not observed between baseline conditional probability and high conditional probability. That is, when low conditional probability trials were excluded from the analysis, there was no interaction between number of stimuli and conditional probability, *F*_(3, 51)_ = 1.160, *p* = 0.330.

The perseverance of this effect when color priming was reduced, but not when conditional probability was removed, indicates that probability alone drives the observed effects on visual search efficiency.

We also replicated our linear modeling from Experiment 1 on the data from Experiment 3. Model 1 (number of cued-color distractors as the only predictor) yields *R*^2^ = 0.104, *F*_(1, 3910)_ = 454.5, *p* < 0.001, while model 2 yields *R*^2^ = 0.123, *F*_(2, 3909)_ = 274.2, *p* < 0.001. Again, testing the nested models confirms that the improvement in fit is significant, *F*_(1, 3909)_ = 84.177, *p* < 0.001. Table 2 shows the intercepts and coefficients for each model.

**Table 2:** **Intercepts and coefficients for the models generated by simple linear regression on the data from the *0.9 conditional probability* condition in Experiment 3**.

**Model 1: rt = α +β_1_(number cued − color stimuli)**					
	**Value**	***SE***	***df***	***t***	**Significance**
α	0.878	0.068	3910	12.94	*p* < 0.001
β_1_	0.195	0.009	3910	21.32	*p* < 0.001
**Model 2: rt = α + β_1_(number cued − color stimuli) + β_2_(number uncued − color stimuli)**					
α	0.726	0.069	3909	10.492	*p* < 0.001
β_1_	0.122	0.012	3909	10.105	*p* < 0.001
β_2_	0.111	0.012	3909	9.175	*p* < 0.001

**Figure 6:**
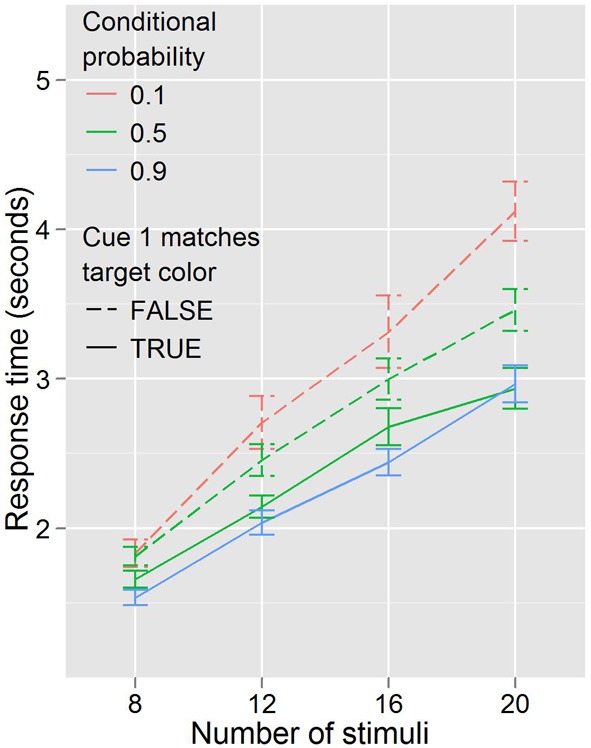
**Response times by distractor number for each of the three conditional probabilities in Experiment 3**. Cue 1 never matched the target color when conditional probability was 0.1, always matched when conditional probability was 0.9, and matched half the time when conditional probability was 0.5.

In this experiment, the baseline condition is not a product of conflicting information from cue 1 and cue 2. Rather, one color or the other is cued, after which the searcher receives information about the validity of this information from the purely symbolic second cue. In contrast to Experiment 1, where it is unclear what the motivation or mechanism might be for selecting or acting based on one cue over the other, here it seems quite possible that searchers might disregard the actual validity of the second (symbolic) cue entirely, and search as if the first (color) cue was always valid. Instead, or in addition, there might be a degree of automaticity to the high conditional probability search behaviors which participants might be unable to completely suppress when predictive and non-predictive cues are interspersed. Figure 6 illustrates how performance differed based on this distinction.

Congruity of the first cue with the target color affects search behavior, even when that cue is indicated to be non-predictive. When conditional probability is held constant (at 0.5) participants are faster to respond when the target color matches the color of the (non-predictive) first cue, *F*_(1, 17)_ = 24.834, *p* < 0.001, and trend toward being more efficient, *F*_(3, 51)_ = 2.630, *p* = 0.060. When cue 1 color matches target color, search trends toward being faster for high conditional probability than for the baseline conditional probability, *F*_(1, 17)_ = 3.495, *p* = 0.079, but there is little evidence that it becomes more efficient, *F*_(3, 51)_ = 1.301, *p* = 0.284; when cue 1 color does not match target color, search is slower, *F*_(1, 17)_ = 5.558, *p* < 0.05, and less efficient, *F*_(3, 51)_ = 3.050, *p* < 0.05, for low conditional probability compared to the baseline conditional probability.

Both possibilities outlined above would be expected to produce these effects: fast RTs for high conditional probability, slow RTs for low conditional probability, and a mix of fast and slow RTs in the baseline condition, depending on the validity of the (non-predictive) color cue. Within this paradigm, it is difficult to differentiate the perception-driven explanation (simplification of the cuing) and the action-driven explanation (inability to suppress automatic search behaviors). However, this distinction, while intriguing, is of secondary interest in this experiment.

Having established that conditional probability modulates visual search efficiency we wondered if this modulation depends on explicit knowledge of conditional probability information. How much knowledge of conditional probability could be learned or deduced simply through the act of searching? Experiment 4 investigates these questions by varying amount and quality of information provided to participants about the conditional probabilities in the task. Essentially, Experiment 1 was repeated, but there were three different sets of participant instructions. Participants in the full information condition received a full description of the relationship between cues and target color (this condition was an exact replication of Experiment 1, and participants received identical information and instructions). Participants in the no information condition were not informed of the relationship between cues and target color. Participants in the misleading information condition were told explicitly (and incorrectly) that there was no relationship between the cues and target color.

**Figure 7:**
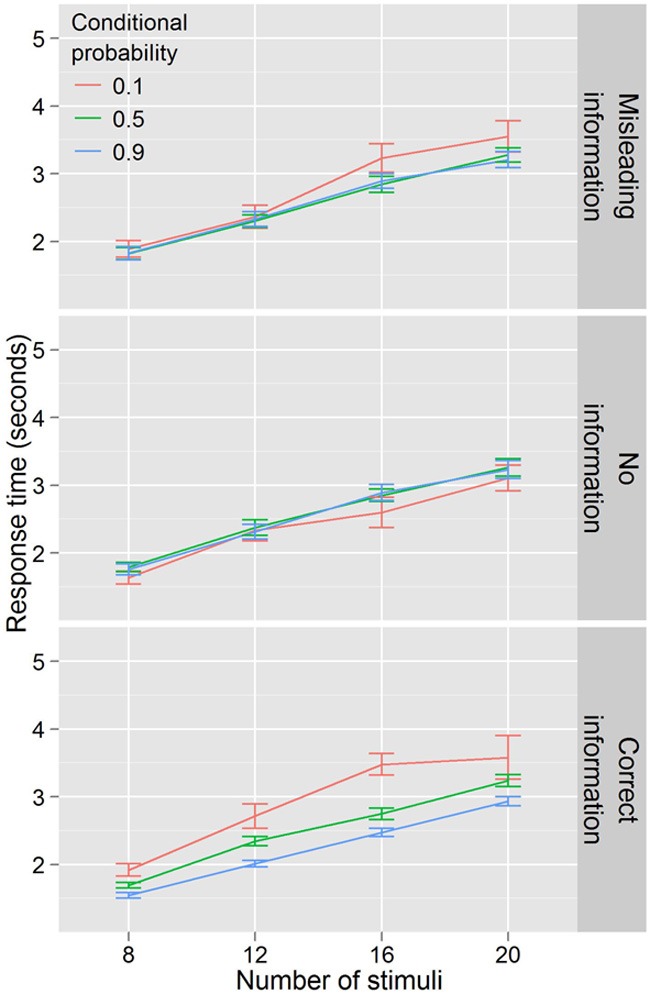
**Response times by distractor number for Experiment 4**. Conditional probability modulates search efficiency only when participants have explicit knowledge.

Figure 7 illustrates the results of Experiment 4. Data in this experiment were analyzed by conducting an ANOVA with RT as the dependent variable, conditional probability (3 levels: 0.1, 0.5, 0.9) and number of stimuli (4 levels: 8, 12, 16, 20) as within-subjects factors, and information condition (3 levels: full information, no information, misleading information) as a between-subjects factor. Again, response time increased with number of stimuli, *F*_(3, 167)_ = 398.159, *p* < 0.001. There was a main effect of conditional probability *F*_(2, 110)_ = 10.866, *p* < 0.001 and this effect differed across information conditions *F*_(4, 110)_ = 9.236, *p* < 0.001. There was no effect of conditional probability in the no information condition *F*_(2, 34)_ = 1.751, *p* = 0.188. In the misleading information condition, there was an effect of conditional probability *F*_(2, 38)_ = 3.543, *p* < 0.05, but only for the low conditional probability targets, which participants were slower to locate. However, in the correct information condition, the pattern from Experiment 1 was repeated, with faster search RTs for high conditional probability and slower RTs for low conditional probability, *F*_(2, 38)_ = 17.429, *p* < 0.001. Evidence for an efficiency component to these differences was present in this condition as well, with a trending number for the stimuli by conditional probability interaction, *F*_(6, 114)_ = 2.019, *p* = 0.069.

RT benefits from learning the probabilities in the task would be expected to take time to emerge in the uninformed and misinformed conditions. Although there was an effect of block in both conditions, *F*_(4, 72)_ = 3.148, *p* < 0.05 for uninformed and *F*_(4, 72)_ = 2.588, *p* < 0.05 for misinformed, there was no block by conditional probability interaction in either condition, *F*_(8, 136)_ = 1.474, *p* = 0.172 (uninformed) and *F*_(8, 144)_ = 1.320, *p* = 0.238 (misinformed). On this basis, we conclude that there was no learning of the probability information in either condition for this task over the roughly 1 h participants spent in the laboratory.

Since the full information condition is an exact replication of Experiment 1, we replicate our linear modeling analysis from Experiments 1 and 3 on these data. As in Experiments 1 and 3, we generate a model 1 which includes only the number of cued-color stimuli term, and a model 2 which includes terms for both number of cued-color stimuli and number of uncued-color stimuli. Model 1 yields *R*^2^ = 0.107, *F*_(1, 4430)_ = 527.9, *p* < 0.001, while model 2 yields *R*^2^ = 0.127, *F*_(2, 4429)_ = 322.8, *p* < 0.001. Goodness of fit comparisons of the nested models confirms that the improvement in fit is significant, *F*_(1, 4429)_ = 105.36, *p* < 0.001. Table 3 details the intercepts and coefficients for each model.

**Table 3:** **Intercepts and coefficients for the models generated by simple linear regression on the data from the *full information, 0.9 conditional probability* condition in Experiment 4**.

**Model 1: rt = α + β_1_(number cued − color stimuli)**					
	**Value**	***SE***	***df***	***t***	**Significance**
α	0.891	0.062	4430	14.28	*p* < 0.001
β_1_	0.193	0.008	4430	22.98	*p* < 0.001
**Model 2: rt = α + β_1_(number cued − color stimuli) + β_2_ (number uncued − color stimuli)**					
α	0.749	0.089	4429	11.84	*p* < 0.001
β_1_	0.115	0.011	4429	10.30	*p* < 0.001
β_2_	0.114	0.011	4429	10.26	*p* < 0.001

It is notable that, in the misleading information condition, participants are slower to locate and report low conditional probability targets, but no faster to report higher conditional probability targets (both compared to the baseline conditional probability condition). Not only did participants perform differently in response to differences in conditional probability that they had been explicitly told did not exist, but the RT cost for low conditional probability targets produced no corresponding benefit for high conditional probability targets.

We believe it likely that instead of providing evidence against the existence of conditional probabilities in the task (as they were meant to), our explicit instructions to participants that cue colors had no relation to target colors paradoxically primed them to explore or perceive exactly such a relationship. Two observations motivate this assertion. First, several participants were suspicious of the spontaneous instruction that there was no relationship between cues and target color (though no participants reported entertaining or acting upon such suspicions at debriefing). Such suspicions about the nature of the task may have led them to engage in exploratory behavior, which could result in the atypical “cost without benefit” pattern of results in the condition as a whole.

Second, our (null) results in the no information condition suggest that participants are at floor performance for learning the statistics of the task when told nothing about those statistics. Because one could suspect that the implicit learning of probability relations might be subtle, we doubled the number of participants for the three conditions of this experiment over what we had used for Experiments 1 and 2. This gave us greater power to detect significant differences in Experiment 4, and makes the negative results relatively more secure. Changes in conditional probability effects between the no information and misleading information conditions correspond to changes in learning and/or acting upon the statistics of the task. Since participants are at floor performance when given no information, such changes must logically be in the positive direction. On the basis of this evidence, we conclude that our misleading information condition actually functioned as a cue that there was a relationship between the cues and target color to be discovered. If a fourth condition were to be implemented with exactly this “partial” information, we would expect to see results similar to those in the misleading information condition.

Overall, these results suggest that explicit knowledge of the probability relationships between cues and target color is necessary for conditional probability to facilitate search performance at the time scale (about 40 min) and number of trials used here. These data do not directly address whether this information would be learned implicitly if more time or trials were allowed, although we speculate, based on results from the misleading information condition, that such implicit learning is possible.

## General discussion

In Experiments 1–3, we demonstrated that participants cued to the probable color of the target in a visual search task search more efficiently for high probability targets and less efficiently for low probability targets; we also showed that participants did not employ a strategy of exhaustively searching high probability stimuli before searching any low probability stimuli. In Experiment 4, we replicated our earlier results showing that participants were faster to locate high conditional probability targets, but only when they were explicitly informed of the probability relationships between cues and targets. When uninformed about the cue-target relationships, participants demonstrated no ability to learn these relationships or to effectively make implicit use of them; when misled about the cue-target relationships, participants showed some sensitivity to them, but were unable to make use of them to facilitate their search.

Previous work, much of which we review in the introduction, has been based on search displays which are simple (e.g., distractor-less searches), regular (e.g., with stimuli always appearing in fixed locations, often in a grid arrangement), or both (e.g., Posner-style tasks in which only one or two stimuli appear in fixed locations). Such experiments can provide evidence for the effectiveness of probability as a graded attentional cue, but do not necessarily generalize beyond the limited search types or the display structures they employ.

Our results provide evidence that people can make use of probability information even under conditions of greater complexity and variation. Despite conditions of near-total randomness for the spatial positions of the target and distractors, and despite significant trial-to-trial variation of distractor number and distractor color ratio (congruent: incongruent), participants in our task made use of conditional probabilities to effectively guide search. What's more, the modulation of efficiency we observe is not a result of a wholesale change in search strategy, but rather is the graded change that would be expected of a probability manipulation. Not only can probability guide search, but it does so even when constraints on complexity and spatial relations are abolished.

We interpret our results as demonstrating that cues can manipulate participants' estimates of the likelihood of forthcoming target features—in our case: color—and not that they bias participant expectations. The distinction between probabilistic biases and expectations is subtle, but important. As reviewed in Summerfield and Egner (2009), this distinction is often ignored or conflated in many studies on attention and the effect of informative cues. Summerfield and Egner (2009) describe two main effects of participant expectations. First, violations of expectations may direct participants to prioritize inspection and evaluate preferentially surprising locations or objects. Second, expectations may bias the interpretations of sensory information. Our protocol did not examine either of these sorts of effects. While our participants did expect, in the colloquial sense, that a magenta target would follow two magenta cues, this only served, in the framework of Summerfield and Egner (2009) to bias attention. In our task, the search arrays always mixed two colors of items in roughly equal proportions. While participants could expect the target to be of a certain color, the appearance of the search array itself provided no opportunity to violate this expectation and therefore no opportunity to prioritize some locations or elements over others on the basis of such a violation. The other function of expectation, to bias interpretation, was also not assessed in our task. Our targets were all identified by virtue of a missing corner. Our participants had no expectation over which corner would be missing, so there was no information available to them that could influence their interpretation of potential targets. Had we used a search array in which items were variously colored between the extremes of magenta and cyan, or if we had used cues that gave information as to a target's missing corner, we might have seen expectation effects, but we did not use such stimuli or cues. In short, the cues we used gave information about the likely color of the target, and as such allow us to interpret our results as probabilistic cues influencing attentional prioritization.

We have presented evidence that probability guides search; however, we find virtually no evidence that participants in this task can learn the conditional probability relationships through exposure alone, something that might be better characterized as implicit statistical learning to distinguish it from our explicit probabilistic cuing. We found this surprising for two reasons. First, the relation between cue color and target color is simple and straightforward—more cues of a certain color predict higher probability for the target to take that color. Second, as highlighted in the opening of this paper, there is a preponderance of evidence in support of the ubiquity and automaticity of visual statistical learning. To conclude this paper, we will highlight some of this relevant evidence and how it might relate to our own results.

One explanation for the inability of uninformed participants to learn the relationships of the cues to targets may be the complexity of the task. There are two cues, not one; we changed the shape and position of the second cue in order to increase the chance that it is noticed as distinct and attended. However, these changes were irrelevant for the cue-target contingency. In such a complex situation, it may be that greater experience than we gave here is necessary to discover the relevant contingencies. This relates to another possible explanation for the inability of our uninformed participants to learn the conditional probability relations between cues: the non-adjacency of the visual presentation. The cues are presented at the beginning of the task, and the task ends when the target is located; a demanding (in terms of visual resources) search interrupts the sequential perception of the statistically related cues and target. Turk-Browne et al. (2005) investigated statistical learning using a sequential presentation task in which shape stimuli were presented in an attended color and an unattended color, and found that nonadjacent relations were learned only for stimuli in the attended color. From this, they concluded that statistical learning is gated by selective attention. Pacton and Perruchet (2008) employed a task in which participants viewed a sequence of digits and performed an arithmetic operation on a pair of digits either immediately succeeding (adjacent pair) or surrounding (nonadjacent pair) a target digit. Their findings and conclusions were similar to Turk-Browne et al.; they found that statistical relationships were only learned for digit pairs that were necessary for task completion, and they concluded that joint processing was necessary for the learning of such dependencies.

Our results are in general agreement with this basic idea, though the term “selective attention” might be too narrow. To complete our task, it was obviously necessary to perceive and attend to the target. Our participants also certainly perceived the cues; each cue was presented alone on the screen for a full second. However, there were no competing stimuli that would require our cues to be selectively attended in the binary sense implied by Turk-Browne et al. Perhaps a more descriptive requirement for implicit learning of statistical relationships would be that the related stimuli be effortfully processed or interacted with. Uninformed participants in our task had little incentive to process or interact with the cues beyond their inevitable passive perception; thus, it is not surprising to us that they fail to discover the connection between cues and target.

Ono et al. (2005) investigated statistical learning in search using a paradigm quite similar to Chun and Jiang (1998). They repeatedly presented pairs of trials in which target position on the current trial was cued by the target position, distractor configuration, or a combination of both on the previous trial. They found that when all features of a previous trial were held constant (target position on no distractor trials, distractor configuration on targetless trials, and both target position and distractor configuration on target present trials with distractors) participants learned the relation between the previous trial's characteristics and the location of the target on the current trial. However, when any aspect of the previous trial was allowed to vary (resulting in one predictive feature and one random feature) learning was abolished. Ono et al. explain these results using a signal: noise framework; predictive features generate signal, random features generate noise, and statistical learning requires a minimum signal: noise ratio.

Our data support such an explanation. By the above definition, there is a great deal of noise inherent in our paradigm. Half of our trials (the 0.5 conditional probability condition) feature non-predictive cues, and every trial involves a search of some length that can also be considered as noise (since the distractors have no predictive value). This explanation also aligns with the selective attention/joint processing account of implicit statistical learning outlined earlier: selectively attending or processing a certain subset of stimuli can be equated to enhancing the signal of the processed set while simultaneously filtering out the noise of the unprocessed set.

We suspect that this is the crux of our participants' inability to learn the cue/target relations in our task. In the uninformed condition, the cues are the aspect of the display least salient to completion of the task; this could lead to a reduction in signal and even, possibly, partial filtering of them as noise. Conversely, the search display itself, the “real” noise (as far as probability learning is concerned), requires the most intensive processing to complete the search task.

It is also possible that the difficulty our participants experienced in learning the probability relations in our task is a consequence of those probabilities dealing with information about a target feature, and not target location. Although there is some evidence to the contrary (Chun and Jiang, 1999) the preponderance of evidence suggests that spatial information enjoys a significant advantage in the realm of implicit statistical learning. For example, Endo and Takeda (2004) showed that contextual cuing effects disappeared when the cued information was changed from spatial position to target identity. In their task involving four clusters of stimuli, each containing different numbers of potential targets, Williams et al. (2009) showed that spatial differences between clusters of stimuli (in terms of likelihood to contain the target) were detected and leveraged quickly and automatically. Surprisingly, participants in their task did not implement search strategies based on features, even though such strategies would be expected to yield faster RTs than the spatial probability-based search patterns that they were quick to adopt.

Our own work highlights this divide between implicit statistical learning of spatial vs. feature information. Specifying and quantifying the conditions under which statistical or implicit learning occurs is an ongoing challenge in the field. Though we have tried to be precise in our reports and discussion and avoid the term “attention,” there is still work to be done in quantifying exactly how search and other tasks like ours can be parsed into measurable quantities such as signal and noise. It is our hope that this paper will be a useful resource to those who work to address this challenge.

### Conflict of interest statement

The authors declare that the research was conducted in the absence of any commercial or financial relationships that could be construed as a potential conflict of interest.
